# Supplementary material for: Norcantharidin Sensitizes Colorectal Cancer Cells to Radiotherapy via Reactive Oxygen Species–DRP1-Mediated Mitochondrial Damage
Source: Antioxidants (Basel). 2024 Mar 14;13(3):347. doi: 10.3390/antiox13030347 (PMC10967768; doi:10.3390/antiox13030347)
Supplement: Supplementary file 1 [file antioxidants-13-00347-s001.zip › Table S1.pdf]

Table S1: shRNA and main primer sequences

Oligo name		
Mfn-1 F：GTGGCAAACAAAGTTTCATGTG	Sangon Biotech	N/A
Mfn-1 R：CACTAAGGCGTTTACTTCATCG	Sangon Biotech	N/A
Mfn-2 F：GTGCTTCTCCCTCAACTATGAC	Sangon Biotech	N/A
Mfn-2 R：ATCCGAGAGAGAAATGGAACTC	Sangon Biotech	N/A
Opa-1 F：TCTGCACACTCAGTTGAAGTAT	Sangon Biotech	N/A
Opa-1 R：GCCTTTGTCATCTTTCTGCAAT	Sangon Biotech	N/A
Drp-1 F：GAGATGGTGTTCAAGAACCAAC	Sangon Biotech	N/A
Drp-1 R：GAGATGGTGTTCAAGAACCAAC	Sangon Biotech	N/A
Fis-1 F：GGAGGAACAGCGGGATTACG	Sangon Biotech	N/A
Fis-1 R：ATGGCCTTGTCAATGAGCCG	Sangon Biotech	N/A
Mff F：AACCCCTGGCACTGAAAACA	Sangon Biotech	N/A
Mff R：AGTCTGCCAACTGCTCGGAT	Sangon Biotech	N/A
GAPDH R: GGTTGTCTCCTGCGACTTCA	Sangon Biotech	N/A
GAPDH F: GGTCCAGGGTTTCTTACTCC	Sangon Biotech	N/A

Table S2: List of primary antibodies and their sources

Antibodies	Source (item number)	WB
Bax	CST 41162	1:1000
Bcl-xL	CST 2764	1:1000
Bim	CST 2933	1:1000
Cleaved-caspase3	CST 9664	1:1000
Survivin	CST 2808	1:1000
Cytochrome C	CST 11940	1:1000
CyclinB1	CST 12231	1:1000
p-Chk2	CST 82263	1:1000
p-CDC2	CST 4539	1:1000
CHK2	Afinity 6033	1:1000
CDC2	Afinity 6108	1:1000
γ-H2Ax	CST 7631	1:1000
GAPDH	CST 5174	1:5000
P21	CST 2947	1:1000
P16	CST 80772	1:1000
Mitofusin-2	CST 11925	1:1000
DRP1	CST 8570	1:1000

p-DRP1	CST 3455	1:1000
Mitofusin-1	CST 14739	1:1000
OPA1	CST 67589	1:1000
MFF	CST 84580	1:1000
