# Supplementary figures and images for: Norcantharidin Sensitizes Colorectal Cancer Cells to Radiotherapy via Reactive Oxygen Species–DRP1-Mediated Mitochondrial Damage
Source: Antioxidants (Basel). 2024 Mar 14;13(3):347. doi: 10.3390/antiox13030347 (PMC10967768; doi:10.3390/antiox13030347)

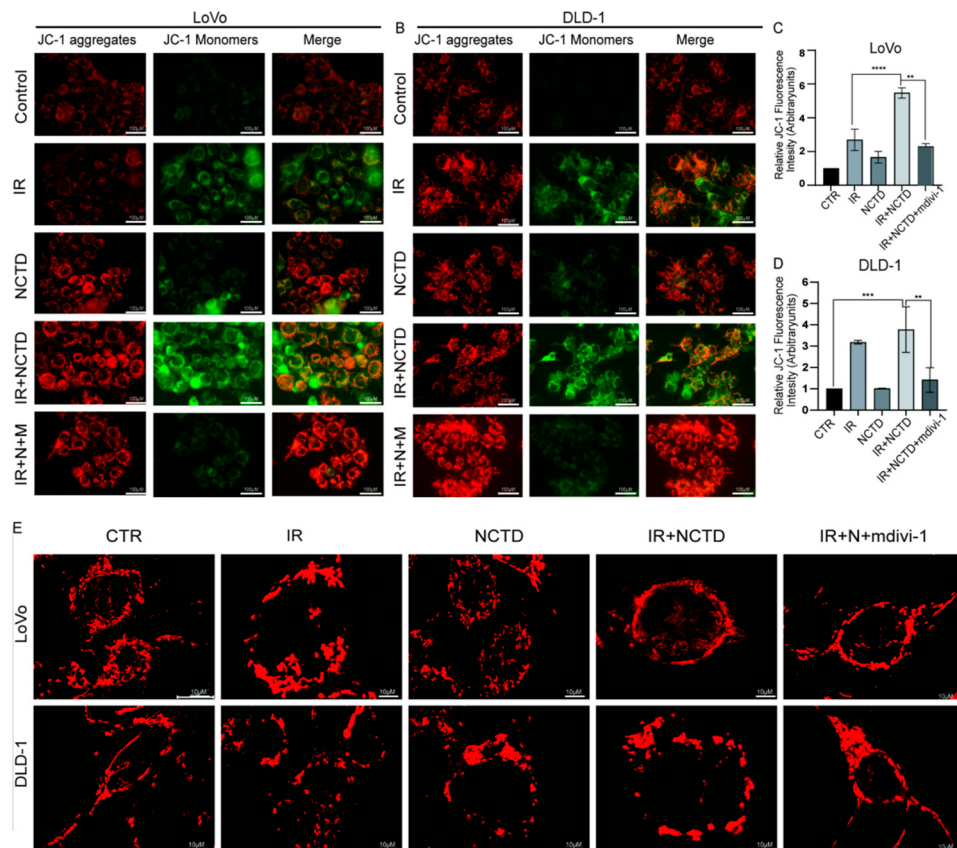

Supplement: Supplementary file 1 [file antioxidants-13-00347-s001.zip › Figure s1.pdf]
